# Interaction of Carbon Dots from Grilled Spanish Mackerel with Human Serum Albumin, γ-Globulin and Fibrinogen

**DOI:** 10.3390/foods10102336

**Published:** 2021-09-30

**Authors:** Guoxin Cui, Yukun Song, Kangjing Liu, Mingqian Tan

**Affiliations:** 1 Academy of Food Interdisciplinary Science, School of Food Science and Technology, Dalian Polytechnic University, Dalian 116034, China; cuiguoxin89@163.com (G.C.); songyukun@126.com (Y.S.); kangjingliu123@163.com (K.L.); 2 National Engineering Research Center of Seafood, Dalian Polytechnic University, Dalian 116034, China; 3 Collaborative Innovation Center of Seafood Deep Processing, Dalian Polytechnic University, Dalian 116034, China

**Keywords:** food-derived carbon dots, human serum albumin, γ-globulin, fibrinogen, interaction

## Abstract

The potential biological effects of food-borne carbon dots (FCDs) generated during food heating procedures on human health has received great attention. The FCDs will be inevitably exposed to blood proteins along with our daily diet to produce unknown biological effects. In this study, the interaction between FCDs extracted from grilled Spanish mackerel and three main types of human plasma proteins including human serum albumin (HSA), human γ-globulin (HGG) and human fibrinogen (HF) was reported. It was found that the grilled Spanish mackerel FCDs could affect the morphology, size and surface electrical properties of the three proteins. The interaction between the FCDs and proteins had different effects on the secondary structure of the three proteins through a static mechanism. The tested HSA, HGG, and HF could adsorb FCDs to reach saturation state within 0.5 min after the adsorption happened. The binding affinity of the FCDs to the plasma proteins was sorted as follows: HF > HGG > HSA. The results of FCDs interacted with plasma proteins provided useful information in the assessment of the safety of FCDs in our daily diet.

## 1. Introduction

The impact of nanoparticles produced during food thermal processing on human health has received considered attention in the research field of food safety [1]. When nanoparticles enter the human body with food, they can inevitably interact with epithelial cells in the gastrointestinal tract, pass through the mucosa, enter the capillaries and distribute in organs and tissues [2]. In this process they will spontaneously adsorb proteins within the plasma, forming one or more layers of corona-like structures, which are generally called protein corona [3,4]. Grilled Spanish mackerel has become a favorite food for consumers because of its unique flavor and comprehensive nutrition. In previous research, food-derived carbon dots (FCDs) found in roast salmon and roast squid could enter the blood of mice after oral administration, and move to various organs such as heart, liver, lungs, and brain through bloodstream [5,6]. Therefore, the FCDs derived from grilled Spanish mackerel are inevitably exposed to plasma proteins after oral administration. However, the interaction of FCDs from grilled Spanish mackerel with various human plasma proteins is unknown. To this end, exploring the possible physiological interaction between FCDs and human plasma proteins is necessary to evaluate the safety of FCDs in baked food products.

It has been reported that the FCDs derived from grilled salmon could combine with digestive proteases (pepsin, trypsin) through the static interaction mechanism, change the secondary structure of the two proteases and inhibit enzyme activity [5]. Human serum albumin (HSA) could reduce the cytotoxicity of FCDs in grilled salmon, reduce the rate of apoptosis and slow down the disorders of glucose and lipid metabolism [7]. Moreover, the FCDs from roast beef could combine with HSA through electrostatic interaction or hydrophobic force, and were distributed in cell lysosomes. HSA also reduced the cytotoxicity of FCDs from beef, inhibited the swelling of the endoplasmic reticulum, caused the decrease of mitochondrial membrane potential, alleviated the generation of reactive oxygen species and slowed down the consumption of glutathione [8]. These findings provided useful information for the combination principle and cell safety of FCDs from different processed foods, which were helpful in the preliminary assessment of the safety of FCDs in our daily diet.

As we know, the blood transports nutrients and oxygen to and takes trash away from our body, which consists of more than 10,000 types of proteins [9,10]. The three most abundant proteins in human blood are albumin (55%), globulin (38%) and fibrinogen (7%) [11]. Albumin can bind and transport water, cations, hormones, fatty acids and drugs, etc., and plays an important role in maintaining the osmotic pressure [12]. Globulins are classified into α1-globulin, α2-globulin, β-globulin and γ-globulin [13]. Among them, human γ-globulin (HGG) includes immunoglobulin, which plays a crucial part in the balance of the immune system and can temporarily enhance the immunity of patients by intravenous injection to treat certain immune diseases such as thrombocytopenic purpura [14,15]. Human fibrinogen (HF) is a glycoprotein that can be converted into fibrin clots by thrombin during vascular injury. The increased content of fibrinogen is considered to be the cause of thrombosis and vascular injury [16]. All these three types of human protein have important functions during human physiological processes.

In this research, the plasma HSA, HGG and HF were selected as model proteins for the research of interaction with the FCDs from grilled Spanish mackerel (*Scomberomorus commerson*) due to their abundant content and important functions. The properties including morphology, size, molecular weight, and fluorescence properties of FCDs were studied, and then the influence of FCDs on fluorescence properties of HSA, HGG and HF was explored using the fluorescence, ultraviolet-visible (UV-Vis) spectroscopy and time-resolved fluorescence spectroscopy, etc, and the combination principle between FCDs and proteins were discussed. Fourier transform infrared spectroscopy (FTIR) and circular dichroism (CD) were used to analyze the influence of FCDs on the structure of the three types of proteins, and the structural changes of HSA, HGG and HF were proved through visualization. The adsorption capacity of FCDs and the three types of proteins were evaluated by the adsorption capacity experiment. The results of the interaction of FCDs with three human plasma proteins can provide useful information for evaluating the bio-effects of FCDs.

## 2. Materials and Methods

### 2.1. Materials

Fresh Spanish mackerel (*Scomberomorus commerson*) was obtained at the fish market in Ganjingzi District, Dalian, China. HSA (96–99%) and PBS buffer solution (0.01 M) was purchased from Solarbio Technology (Beijing, China). HGG (≥99%) and HF (50–70%, ≥80%, clottable) were obtained from Sigma Aldrich (Shanghai, China). The bicinchoninic acid (BCA) protein assay kit was obtained from Beyotime Biotechnology (Shanghai, China). All other reagents applied in this research were analytically pure.

### 2.2. Characterization Methods

The molecular weight of FCDs was measured by matrix-assisted laser desorption/ionization time-of-flight mass spectrometer (MALDI-TOF-MS, Autoflex III, Bruker Daltonics, Bremen, Germany), using 2, 5-dihydroxybenzoic acid as a matrix. Transmission electron microscope (TEM) analysis was used a TEM (JEM-2100, JEOL, Tokyo, Japan) at a voltage of 200 kV. The ultrasonic particle size analyzer (DT1200) was used to measure the ζ-potential of the samples. UV-Vis spectrophotometer (Lambda 35, PerkinElmer, Norwalk, CT, USA) was used to record the absorption spectra. The fluorescence spectra were performed with the F-2700 spectrofluorometer (Hitachi, Tokyo, Japan). The fluorescence lifetime was ascertained by an FLS980 spectrometer (Edinburgh Instruments, Edinburgh, UK). FTIR was conducted by a PerkinElmer spectrometer (Frontier, Norwalk, CT, USA). CD spectra were performed by a JASCO circular dichroism spectrometer (J-1500, Tokyo, Japan). Atomic force microscope (AFM) was used to measure the two/three-dimensional morphology and height of the samples (AFM-5500M, Hitachi, Tokyo, Japan). Unless otherwise stated, the sample concentration was 1 mg/mL. All samples were dissolved in PBS buffer (0.01 M, pH 7.2–7.4). Before the test, the FCDs and protein mixture liquid was incubated in a water bath at 37 °C for 10 min.

### 2.3. Preparation of FCDs

The extraction method of FCDs was referred to our previous reporting [5]. Briefly, the fresh Spanish mackerel meat (1500 g, wet weight) was cut into pieces of 2 cm × 1 cm × 1 cm, and grilled at 230 °C for 30 min. The roasted flesh (405 g) was added to ethanol in a ratio of 1:5 (*w*/*v*), sonicated for 2 h, and the mixture was filtered to remove impurities. The concentrated ethanol solution was then added to 150 mL of deionized water, and extracted with dichloromethane three times to remove the hydrophobic phase. The aqueous phase was centrifuged at 10,000 rpm for 10 min to remove the precipitate. The supernatant was concentrated by vacuum rotary evaporation, and purified by the D101 microporous resin column using deionized water as the eluent. The aqueous solution was concentrated by rotary evaporation again and purified through 0.22 μm filter membrane and semi-preparative high-performance liquid chromatographic column C18 (20 mm × 300 mm, 10 μm) utilizing 10% methanol aqueous solution as mobile phase with the flow velocity of 18 mL/min. The fluorescent fractions were collected, concentrated and lyophilized to obtain FCDs powder (104 mg).

### 2.4. Fluorescence Analysis

The fluorescence data were calculated using the Stern-Volmer equation:

$$\frac{F_{0}}{F}=1+k_{q}\tau_{0}\left[Q\right]=1+K_{sv}\left[Q\right]$$

In this equation, *F*_0_ and *F* are the fluorescence intensity of the protein solution with FCDs not added or added, respectively, *K_q_* is the bimolecular quenching constant, *τ*_0_ is the average life expectancy of proteins, *Κ_sv_* is the dynamic quenching constant, and [*Q*] is the concentration of FCDs. Tryptophan in protein has the strongest fluorescence peak at the excitation wavelength of 280 nm, the maximum excitation wavelength of tyrosine is 275 nm, and phenylalanine also has the fluorescence peak at the excitation wavelength of 280 nm [17]. Therefore, the change of inherent fluorescence properties of protein can be monitored by selecting the excitation wavelength of 280 nm.

Fluorescence lifetime was measured according to our previous method using an FLS980 spectrometer (Edinburgh Instruments, Edinburgh, UK) [18].

### 2.5. Adsorption Measurement

A total of 5 mL FCDs solution (2.0 mg/mL) was mingled with 5 mL of HSA, HGG, and HF (0.5 mg/mL), respectively. The solution had been preheated and incubated in a 37 °C water bath. The reaction solution (0.5 mL) was collected at the corresponding time point at 0, 0.5, 1, 2, 3, 4, 5, 10, 20, 30, 40, 50, 60, 120, 180, 240 min, which was injected into 4 °C PBS buffer (pH 7.2–7.4) and diluted 5 times to stop the interaction. The protein-FCDs complex was centrifuged through an ultrafiltration centrifuge tube (50 k MWCO) at 3600 rpm, 4 °C for 10 min, to remove the free FCDs. The ultrafiltrate was collected and tested with 10 μL of Bradford solution to check whether there was protein leakage in the filtrate. The concentration of FCDs was measured at the wavelength of 562 nm using the BCA kit (Solarbio). The concentration of FCDs was quantified by standard curve, and the concentration of FCDs adsorbed by proteins was calculated by the difference between the initial concentration that was added and measured in the ultrafiltrate.

## 3. Results and Discussion

### 3.1. Characterization of FCDs

**Figure 1:**
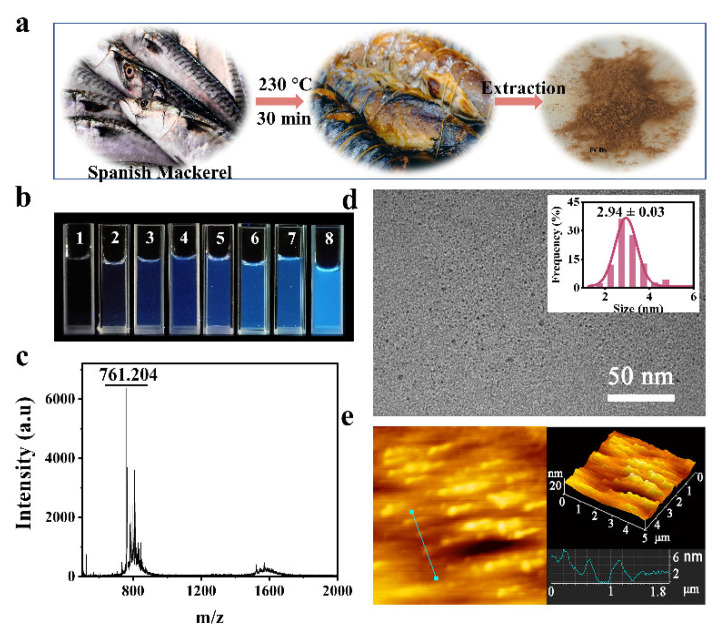
Characterization of FCDs. (**a**) Schematic diagram of FCDs derived from grilled Spanish mackerel at 230 °C for 30 min. (**b**) FCDs aqueous solution under 365 nm ultraviolet light. The concentrations of FCDs in 1–8 solution were 0, 2, 4, 6, 8, 10, 12, 24 × 10^−5^ mol/L, respectively. (**c**) MALDI-TOF-MS spectrum of FCDs. (**d**) TEM image and the corresponding size analysis of FCDs (*n* = 180). (**e**) Two-dimensional (2D) and 3D AFM images of FCDs derived from grilled Spanish mackerel.

The FCDs, fluorescent carbon-rich nanoparticles with good water solubility, were extracted from grilled Spanish mackerel and appeared as a brown powder (Figure 1a). The aqueous solution of FCDs emitted blue fluorescence under 365 nm ultraviolet light and the fluorescence intensity increased with the increase of the concentration (Figure 1b). The analysis of MALDI-TOF-MS showed a main peak at 761.204 *m*/*z* (Figure 1c), which was composed of carbon-hydrogen-oxygen-nitrogen elements without exact molecular structures. In the subsequent computational process, 761.204 Da was used as the average molecular weight of FCDs. TEM image showed that the FCDs were nearly spherical with uniform size and good dispersion. The average diameter of FCDs was 2.94 ± 0.03 nm (Figure 1d). Besides, the AFM images clearly showed that the height of FCDs in grilled Spanish mackerel was about 2–4 nm (Figure 1e), which further confirmed the TEM results. In another study, the height of FCDs in roast duck was 2–4 nm, and as the roasting temperature increased from 200–300 °C, the height of FCDs became more uniform [6]. The height of FCDs in grilled squid was 2–6 nm, and it was speculated that the height of FCDs was comparable with the size of TEM [19]. The FCDs with an ultra-small size and high surface to area ratio may have great potential interaction with human blood proteins.

### 3.2. Interaction between FCDs and Proteins

**Figure 2:**
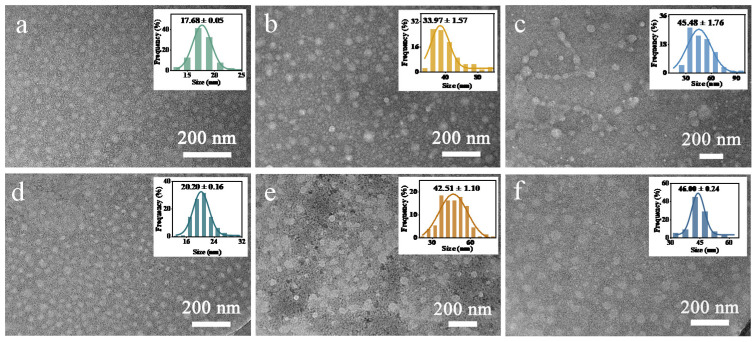
TEM pictures of (**a**) HSA, (**b**) HGG, (**c**) HF (**d**) HSA-FCDs, (**e**) HGG-FCDs, (**f**) HF-FCDs, and the corresponding size distributions measured in 0.01 M PBS.

To explore the interaction effects of FCDs with HSA, HGG, and HF, TEM was used to measure the size, dispersion and morphology of the three types of proteins after interaction with FCDs. The proteins, negatively stained with phosphotungstic acid, showed a nearly spherical morphology. In detail, the TEM image of HSA demonstrated that the HSA was well-dispersed and the average size was 17.68 ± 0.05 nm (Figure 2a). This is similar to the HSA size determined by crystallographic methods at a resolution of 6.0 Å with unit cell size a = b = 186.5 ± 0.5 Å, c = 81.0 ± 0.5 Å [20]. The size also corresponds to the unit cell size determined at a resolution of 3.0 Å, a = b = 187.1 Å, c = 80.5 Å [21]. Actually, the shape of HSA was a heart-like asymmetric structure with equilateral triangle sides of ~80 Å [22]. The observed HSA image was probably the dimer or multimer of the single molecules in the PBS solution. After interacting with FCDs, the average size of HSA increased to 20.20 ± 0.16 nm (Figure 2d), which corresponds to the sum of the sizes of FCDs and HSA, indicating that HSA and FCDs may adsorb each other and form the complex. The average size of HGG was 33.97 ± 1.57 nm (Figure 2b). Kumakura et al. proposed that the size of HGG molecules was 100–200 Å, and the apparent volume would increase due to the entanglement of the molecules in the solution [23]. After interacting with FCDs, the size of HGG and FCDs complex increased to 42.51 ± 1.10 nm (Figure 2e). Unlike HSA and HGG, the HF did not completely exist in its original form, and multimers formed by the horizontal end-to-end connections of particles can be observed (Figure 2c). The high heterogeneity of HF had been confirmed in the previous studies [16,24,25]. The high degree of heterogeneity is inseparable from the intermediates produced in the three steps of converting fibrinogen to fibrin. In the first step, fibrinogen releases fibrin peptides and forms fibrin monomers under the catalysis of thrombin. In the second step, fibrin monomers form fibrin polymers through non-covalent interactions. In the third step, the polymer aggregates to form the fibrin clot [26,27]. Several reports proved that the length of HF was around 45 nm [28,29], and the average particle size here was also 45.48 ± 1.76 nm (Figure 2c). But fibrinogen is a slender bio-polymer, and the size of the particles here could correspond to the fibrinogen monomer of 41.0–43.5 nm (Figure 2c) [30]. After interacting with FCDs, the particle size of the HF slightly increased to 46.00 ± 0.24 nm, and the connection among the particles could not be found in the sample (Figure 2f). It is speculated that the FCDs probably prevent the non-covalent interaction between HF particles, so that the monomers could not be connected, and inhibited the formation of fibrin multimers. All the size increase of the HSA, HGG and HF after interaction with FCDs, indicated that the FCDs caused influence when they were dispersed in the aqueous solution of HSA, HGG and HF, respectively.

**Figure 3:**
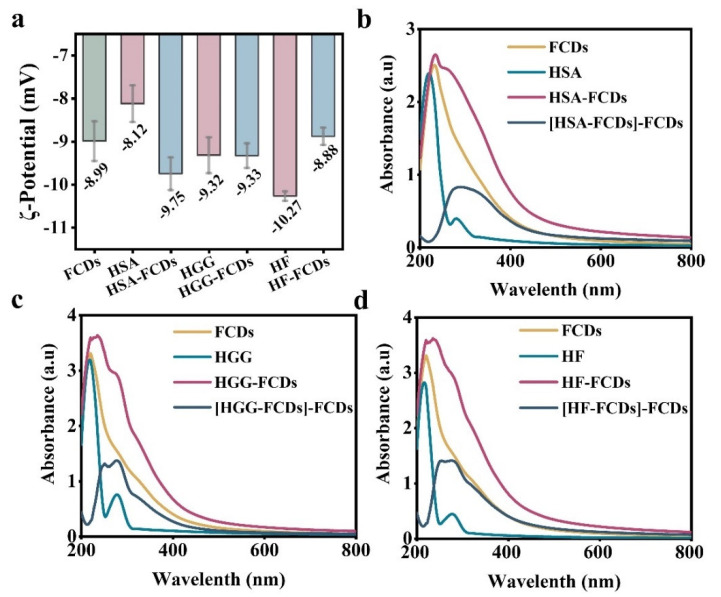
ζ-potential measurement and UV-Vis absorption spectra of the interaction of FCDs with HSA, HGG and HF. (**a**) ζ-potential. *n* = 6. UV-Vis absorption spectra of (**b**) HSA, (**c**) HGG, and (**d**) HF.

Then ζ-potential was monitored to study the change of surface charge of HSA, HGG, HF in the presence of FCDs. The result showed that the average potential of FCDs was −8.99 mV (Figure 3a). After adding FCDs to the protein particles, the average potential of HSA dropped from −8.12 to −9.75 mV, while the average potential of HGG was basically unchanged. The average potential of HF showed an opposite trend to that of HSA, rising from −10.27 to −8.88 mV (Figure 3a). One of the possible explanations for this phenomenon is that the FCDs may change the surface structure of HSA and HF during the formation of HSA-FCDs and HF-FCDs. It should be noted that ζ-potential provided indicative information about colloidal stability, but the steric interaction between molecules also contributed to colloidal stability [31], which might also be used to explain the slight change in the ζ-potential of HGG after interacting with FCDs. In addition, the HF showed an electrical neutralization phenomenon after adding FCDs, suggesting that the interaction between HF and FCDs was related to electrostatic characteristics.

Figure 3b–d show the UV-Vis absorption spectra of FCDs, HSA, HGG and HF, respectively. The spectra of HSA, HSA-FCDs, and [HSA-FCDs]-FCDs were significantly different. After adding FCDs, the single peak of HSA red-shifted and the absorption intensity also increased. Similar results were also found for the HGG and HF. This indicated that the addition of FCDs affected the microenvironment of the three types of protein, and this indicated that the proteins and FCDs adsorbed to each other and formed the complex.

**Figure 4:**
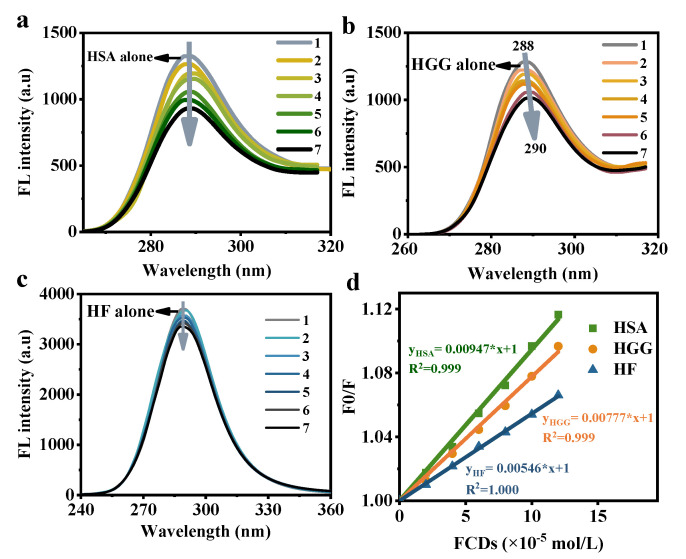
Fluorescence emission spectra of (**a**) HSA, (**b**) HGG, (**c**) HF in the existence of different concentrations of FCDs. (**d**) Stern-Volmer linear relationship curves of HSA, HGG, and HF quenched by FCDs at 298 K. The excitation wavelengths were 280 nm. The concentrations of HSA, HGG and HF in the solution were 1 × 10^−7^ mol/L, and the concentrations of FCDs in the 1–7 curves were 0, 2, 4, 6, 8, 10 and 12 × 10^−5^ mol/L, respectively.

The fluorescence spectra change may provide in-depth information on the molecular interaction between FCDs and plasma proteins. As the concentration of FCDs increased, the fluorescence emission intensity of the proteins gradually decreased (Figure 4a–c). Meanwhile, the maximum emission wavelengths of the HGG spectra were red-shifted, suggesting that the FCDs had a more obvious effect on HGG fluorophores. This was probably due to the increase in the protonation level of the area around the fluorophore, resulting in the change of hydrophobic domain [32]. In the concentration of 0–12 × 10^−5^ mol/L, the fluorescence results were in keeping with the Stern-Volmer equation with good linear relationships between the *F*_0_/*F* value and the FCDs concentration (Figure 4d). The calculated quenching rate constants kq from the Stern-Volmer equation were *k_q_* (HSA) = 9.47 × 10^10^ M^−1^ s^−1^, *k_q_* (HGG) = 7.77 × 10^10^ M^−1^ s^−1^, *k_q_* (HF) = 5.46 × 10^10^ M^−1^ s^−1^, respectively. All these *k_q_* values were larger than the maximal diffusion rate constant of biomacromolecule (2 × 10^10^ M^−1^ s^−1^) [33]. This finding proved that FCDs combined with HSA, HGG and HF through a static quenching mechanism. Static quenching was arisen from the forming process of nonluminous ground state complexes [34], which inferred that the FCDs formed complexes with HSA, HGG, and HF. Rayleigh scattering is considered to be one of the interference factors in fluorescence spectrometry, and its peak resonates with the fluorescence peak. When the particle size is the same, the strength of the resonant scattering peak has been proved to be proportional to the concentration of nanoparticles [35]. Herein, increasing concentration of FCDs was found to quench the intrinsic fluorescence of proteins, without the negative effects of Rayleigh scattering. In addition, unlike dynamical quenching that only influences the excited state of the molecule, static quenching will lead to the change of the absorption spectra of the fluorophore, but does not affect the absorption spectra of the quenched substance [36]. This was demonstrated in the UV-Vis absorption spectra of Figure 3.

**Figure 5:**
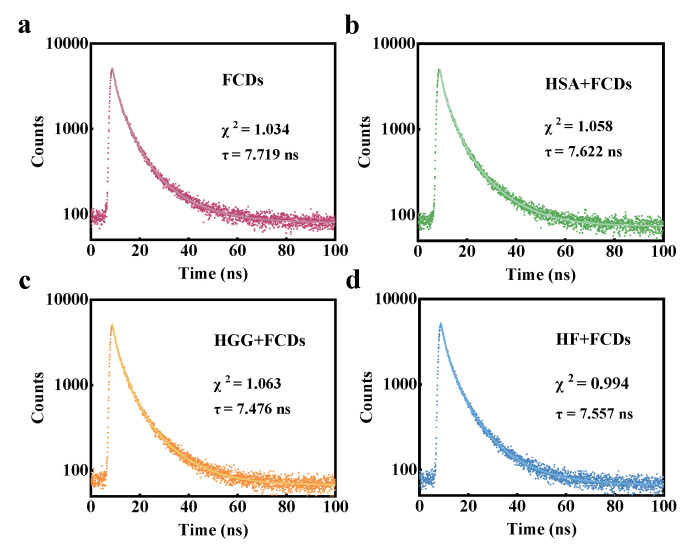
Fluorescence lifetime decay curves of (**a**) FCDs, and (**b**) FCDs-HSA, (**c**) FCDs-HGG and (**d**) FCDs-HF.

Time-resolved fluorescence spectroscopy can verify the combination mechanism by analyzing the fluorescence lifetime of the nanoparticles before and after the interaction with plasma proteins. The fluorescence lifetimes of FCDs calculated by the multi-exponential equation changed from 7.719 to 7.622, 7.476 and 7.557 ns after interacting with HSA, HGG and HF, respectively (Figure 5a–d). The lifetimes were reduced by 1.26, 3.15 and 2.10%, respectively, and the changes in fluorescence lifetime were much lower than the reported dynamic quenching degree of 31.17% [18,37]. This further supported the static combination of the three proteins with FCDs.

**Figure 6:**
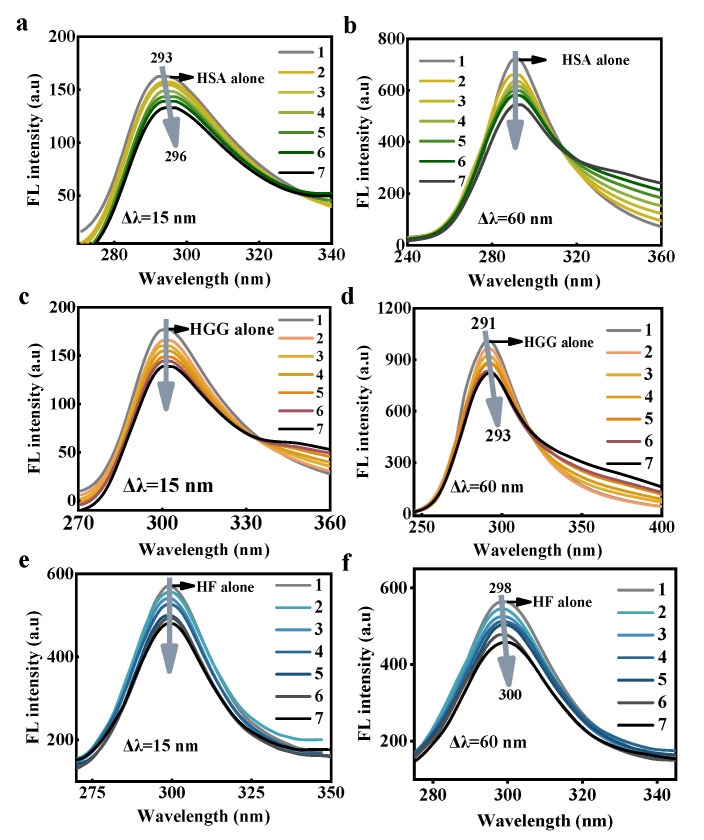
Synchronous fluorescence spectra of HSA, HGG and HF incubated with different concentrations of FCDs under Δλ = 15 and 60 nm. The excitation wavelength was 280 nm. (**a**) HSA under Δλ = 15 nm. (**b**) HSA under Δλ = 60 nm. (**c**) HGG under Δλ = 15 nm. (**d**) HGG under Δλ = 60 nm. (**e**) HF under Δλ = 15 nm. (**f**) HF under Δλ = 60 nm. The concentrations of HSA, HGG and HF in the solution were 1 × 10^−7^ mol/L, and the concentrations of FCDs in the 1–7 curves were 0, 2, 4, 6, 8, 10 and 12 × 10^−5^ mol/L, respectively.

Synchronous fluorescence spectroscopy can provide instruction about the biomolecular microenvironment around the protein or amino acid fluorophores. The feature information of tyrosine (Tyr) and tryptophan (Trp) can be monitored by inputting a fixed Δλ value of 15 or 60 nm between the two monochromators [38,39]. With the concentration increase of FCDs, the synchronous fluorescence intensity of all amino acid residues of the three proteins decreased gradually. The maximum emission wavelength of HSA on the condition that Δλ = 15 nm was found a red-shift (Figure 6a), indicating that the hydrophobicity of Tyr residues decreased. The maximum emission wavelength under Δλ = 60 nm did not change (Figure 6b), indicating that there was little microenvironmental disturbance around Trp [5]. The change of maximum emission wavelengths of HGG and HF was not obvious under Δλ = 15 nm (Figure 6c,e), while the maximum emission shifted to longer wavelength slightly under Δλ = 60 nm (Figure 6d,f), indicating that the residue hydrophilicity and chain extension of HGG and HF increased as well [40].

### 3.3. Structural Analysis

**Figure 7:**
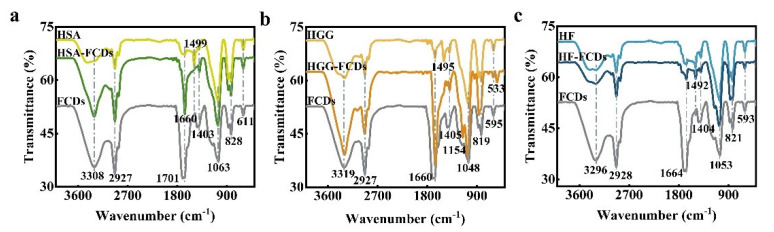
FTIR spectra of FCDs interacting with HSA, HGG, HF. (**a**) HSA. (**b**) HGG. (**c**) HF.

Next, the conformational change of the three proteins after the interaction with FCDs was analyzed with FTIR spectroscopy. The peaks of FCDs at 3308 cm^−1^ and 2927 cm^−1^ were attributed to the stretching vibration of C-OH and C-H, respectively (Figure 7a) [41]. The amide I band in the range of 1600–1700 cm^−1^ corresponded to the C=O tensile vibration, while the amide II band between 1500 and 1600 cm^−1^ was due to the N-H bending vibration [42]. The peaks at 1403 and 1063 cm^−1^ were related to C-N bonds and C-O-C bonds, respectively [6]. After the interaction of HSA with FCDs, the absorption peak of the amide I band was significantly enhanced, while the amide II band was weakened. This indicated that the secondary structure of HSA might be rearranged. After HGG interacted with FCDs, the absorption peak of amide I band was greatly enhanced (Figure 7b), suggesting that the interaction greatly affected the C=O tensile vibration of HGG. After HF interacted with FCDs, the intensity and position of the amide band absorption peak were basically unchanged (Figure 7c). The FTIR spectra showed that the FCDs could affect the structure of the three types of proteins, thus prompting us to further explore the influence of FCDs on the structure of proteins.

**Figure 8:**
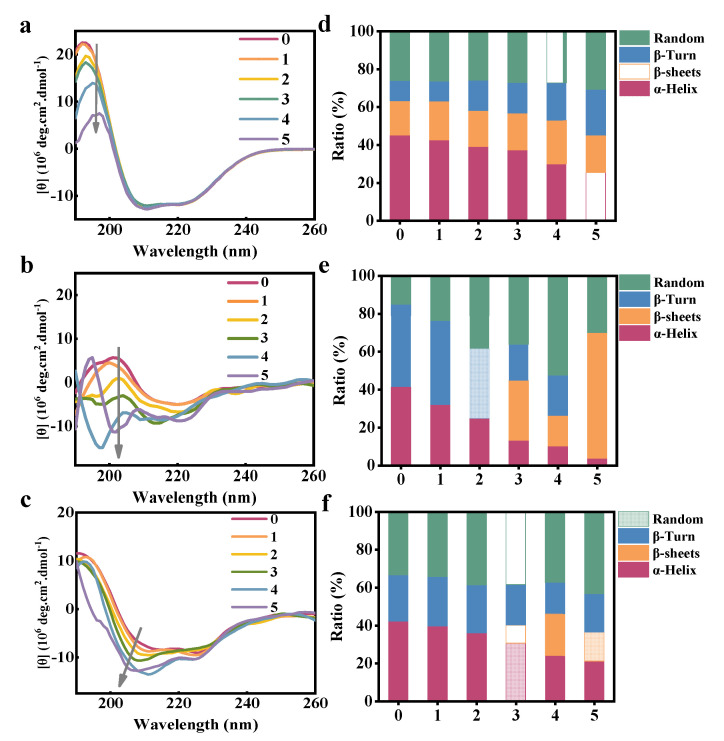
CD spectra of FCDs interacting with HSA, HGG, HF and the corresponding secondary structure ratio analysis. CD spectra of (**a**) HSA, (**b**) HGG, and (**c**) HF. Secondary structure ratio analysis of (**d**) HSA, (**e**) HGG, and (**f**) HF. The concentrations of HSA, HGG and HF in the solution were 1 × 10^−7^ mol/L, and the concentrations of FCDs in the 0–5 curves were 0, 2, 4, 6, 8 and 10 × 10^−5^ mol/L, respectively.

To further explore the effect of FCDs on the secondary conformation of the plasma proteins, CD spectroscopy was applied as a powerful tool to investigate the interaction of FCDs with plasma proteins by measuring the proportion of the four secondary structures of proteins in the total secondary structure. The proteins with different secondary structures have different absorption peaks and absorption intensities. For example, the α-helix has a positive absorption band at 192 nm, with two negative absorption bands at 222 and 208 nm, respectively. The β-sheet has a negative absorption band at 216 nm and a positive absorption band around 195–198 nm. The percentage of protein secondary structures can be calculated based on the absorption intensity of particular secondary structure with the software. As shown in Figure 8, the CD spectra in the range of 190–260 nm showed that the proportion of α-helix of natural HSA and HF were 45.0% and 42.2%, respectively, which decreased to 25.3% and 21.1% when the concentration of FCDs increased from 0 to 1 × 10^−4^ mol/L. The α-helix of natural HGG decreased from 41.3% to 3.7% when the concentration of FCDs increased from 0 to 1 × 10^−4^ mol/L. The ratios of the secondary conformation of the three proteins were listed in Table 1. The β-sheet of HSA remained unchanged, while increased significantly for the HGG and HF with the increase of the FCDs concentration. The β-turn showed an increasing trend from 10.8% to 24.2% for HSA, while it completely disappeared for HGG when the concentration of FCDs increased from 0 to 1 × 10^−4^ mol/L. In addition, β-sheet appeared as the FCDs increased to 6 × 10^−5^ mol/L for HF. So the secondary structure of HGG and HF was affected by the FCDs, and there might be the unfolding of peptides, and even the destruction of the protein hydrogen bond network leading to the distortion of β-sheet [43]. With the increase of the concentration of FCDs added, the proportion of random coil of the three types of proteins showed an increasing trend, which also proved that FCDs caused the structure change of the proteins.

**Figure 9:**
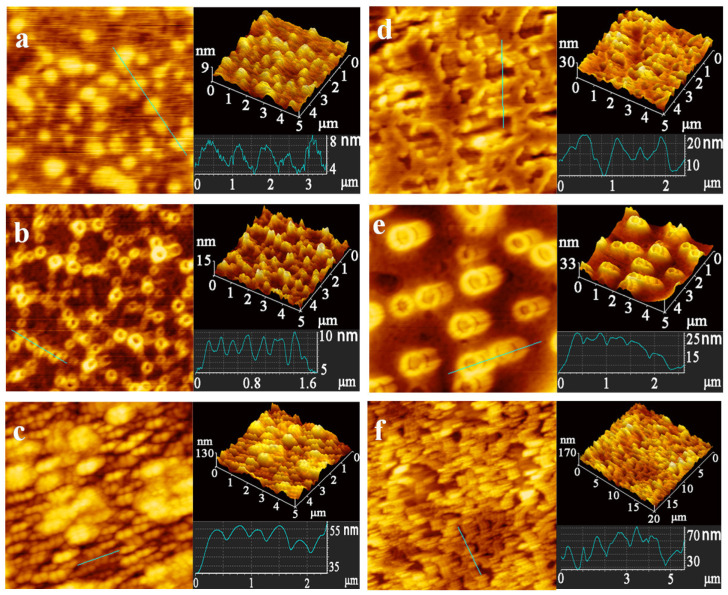
AFM 2D and 3D images of (**a**) HSA, (**b**) HGG, (**c**) HF, (**d**) FCDs-HSA, (**e**) FCDs-HGG, (**f**) FCDs-HF, and the corresponding size and height analysis.

More detailed information on the surface morphology and structure of the protein was obtained by AFM imaging [44]. Figure 9 shows the AFM images of the HSA, HGG and HF before and after interaction with FCDs. The AFM image of HSA showed the nanoparticles with a height of 4–8 nm (Figure 9a), which corresponded to the reported oblate ellipsoid with a heart-shaped structure having a side length of 80 Å described by crystallographic methods at a resolution of 2.8 Å [22,45]. After adding FCDs, the dispersion of HSA-FCDs changed to cluster, and the height increased to 10–15 nm (Figure 9d). This inferred that the HSA and FCDs adsorbed to each other and formed the complex. The AFM image of HGG also showed a spherical shape in the plane diagram. The difference was that the surface center of particles was hollow and uneven, with an average height of 3–6 nm (Figure 9b). Its height can correspond to the HGG measured by the ellipsometer, with an average height of 45 Å [46], and also close to the height of immunoglobulin G (4 nm) [47]. After interacting with FCDs, the height of HGG increased to 9–11 nm, and the obvious adhesion phenomenon was observed (Figure 9e). In addition, the morphology of HF was a slender strip with multiple nodules (Figure 9c), which corresponded exactly to the three-nodular structure described in previous studies [48,49]. The height of HF was 5–15 nm (Figure 9c), the dense particles made the substrate height uneven, which limited the accurate analysis. Evans-Nguyen et al. described the height of the HF monolayer as 5.5 ± 2.2 nm [50]. Kollman et al. determined the minimum three-dimensional crystal size of HF at a resolution of approximately 3.3 Å to be 94.87 Å [51]. Fowler & Erickson described the two outer nodules of HF with a diameter of 6–7 nm and the middle nodule with a diameter of 4–5 nm [52]. The height of HF in the range of 5–15 nm was consistent with the results that mentioned above. After interacting with FCDs, the morphology of HF changed, the nodular structure was changed, and the height increased to 20–30 nm (Figure 9f). This revealed that HF and FCDs adsorbed each other to form nanocomplex. The AFM results directly proved that the FCDs decreased the uniformity of the HGG, and changed the surface structure of HSA and HF.

### 3.4. Adsorption Capacity and Ratio

**Figure 10:**
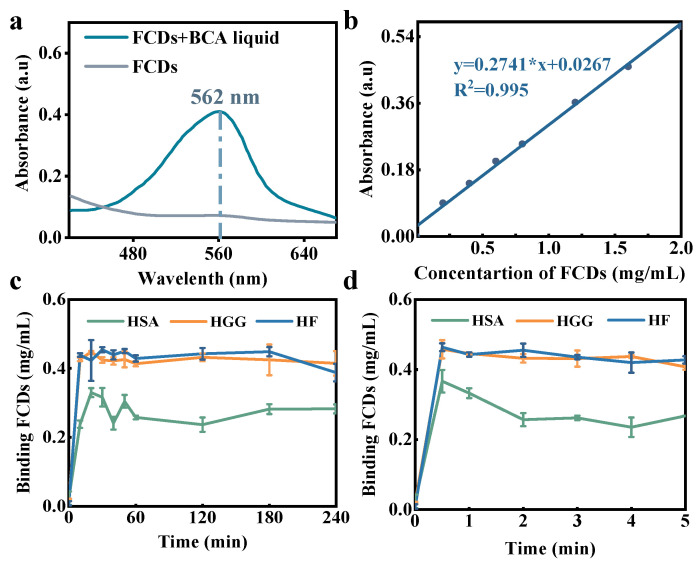
Adsorption capacity experiment of FCDs with HSA, HGG and HF. (**a**) UV-Vis absorption spectra of FCDs and BCA reagents. (**b**) Standard curve of FCDs concentration and absorbance at the ultraviolet wavelength of 562 nm. Binding amount of FCDs by HSA, HGG and HF from (**c**) 0–240 min and (**d**) 0–5 min.

In addition to the effects of FCDs on the structure of the three types of protein, HSA, HGG and HF, the adsorption capacity and binding ratio between proteins and FCDs were also studied. The traditional BCA protein adsorption capacity determination method requires the separation of the complex by gravity sedimentation. However, the FCDs have a similar density to human plasma protein, suggesting that the protein-FCDs cannot be separated from free protein or FCDs by gravity. Therefore, the BCA method was modified based on the principle that FCDs could react specifically with the BCA reagent and the absorbance of the BCA-FCDs was proportional to the concentration (Figure 10a,b). The excess FCDs in the reaction system were separated through 50 kDa ultrafiltration centrifuge tubes to determine the concentration of FCDs adsorbed by plasma proteins. Since both FCDs and proteins could react with BCA reagents, Bradford reagent was used to check the ultrafiltrate to ensure that there was no protein mixing before proceeding to the next step. The results showed that three types of proteins adsorbed high levels of FCDs within 5 min, and the adsorption within 4 h did not significantly change (Figure 10c). Increasing the concentration of FCDs did not increase the adsorption level, indicating that the tested human plasma proteins and FCDs had reached saturated adsorption. The adsorption situation within 5 min was further studied, and it was found that the adsorption of the three types of proteins reached high levels within 0.5 min (Figure 10d). This proved that the interaction between proteins and FCDs happened rapidly, which was probably completed less than 0.5 min that was not detectable using the current method. The interaction between FCDs and protein was stable. Zhang et al. monitored the evolution of the reaction system from 5 min to 5 h, and no time-dependent change in protein binding was found [53]. The protein adsorption behavior was likely to reach equilibrium within 5 min. Tenzer et al. reported that SiO_2_ nanoparticles incubated in human plasma adsorbed more than 300 proteins in a short period (<0.5 min) [54]. Dell’Orco et al. established a mathematical model to describe the kinetic process of protein corona formation based on the association/dissociation rate and also demonstrated protein corona could be formed quickly in a very short time (*t* = 0.05 s), and the complex was relatively stable within the first 20 s [55]. Interestingly, it was also found that the concentration of FCDs adsorbed by the same amount of protein increased with the increase of theoretical molecular weight of the protein (HSA: 66 kDa; HGG: 180 kDa; HF: 240 kDa), the adsorption capacity was sorted as HF > HGG > HSA, which fully demonstrated that the ability of proteins to combine with FCDs depends on the intrinsic properties of proteins.

## 4. Conclusions

The FCDs derived from grilled Spanish mackerel could affect the size, stability and aggregation morphology of HSA, HGG, and HF, resulting in significantly different surface potential changes. The interaction between FCDs and HSA, HGG, and HF changed the secondary structure of the three types of proteins. The FCDs bound to HSA, HGG, and HF through the static mechanism, and the adsorption reaction occurred quickly within 0.5 min. The binding affinity of the FCDs to the plasma proteins was sorted as follows: HF > HGG > HSA. The interaction between FCDs and three types of plasma proteins remains to be further explored to demonstrate the effect on cell uptake, cytotoxicity and cell metabolism.

## Figures and Tables

**Table 1:** Changes in the secondary conformation of HSA, HGG, HF in the presence of FCDs.

Ratio (%)	FCDs (mmol/L)	0.00	0.02	0.04	0.06	0.08	0.10
HSA	α-Helix	45.0	42.6	39.1	37.3	29.9	25.3
	β-sheet	18.2	20.4	19.0	19.4	23.0	19.8
	β-Turn	10.8	10.7	16.0	16.2	19.8	24.2
	Random	26.0	26.2	26.0	27.1	27.2	30.7
HGG	α-Helix	41.3	32.0	24.6	13.1	10.1	3.7
	β-sheet	0.0	0.0	0.0	31.6	16.2	66.3
	β-Turn	43.6	44.3	37.0	19.1	21.2	0.0
	Random	15.1	23.8	38.3	36.2	52.5	30.0
HF	α-Helix	42.2	39.7	36.0	30.6	23.9	21.1
	β-sheet	0.0	0.0	0.0	9.6	22.3	15.4
	β-Turn	24.4	26.0	25.4	21.4	16.6	20.2
	Random	33.4	34.3	38.6	38.3	37.2	43.3

## Data Availability

The datasets generated for this study are available on request to the corresponding author.
